# Five-Day Preoperative Radiation Therapy for Patients With High-Risk Soft Tissue Sarcoma: A Nonrandomized Clinical Trial

**DOI:** 10.1001/jamanetworkopen.2025.50195

**Published:** 2025-12-17

**Authors:** John Nikitas, Joseph K. Kendal, Ricky R. Savjani, Nicholas Jackson, Nicholas Peterson, Jie Deng, Jackie Hernandez, Natalie Chong, Arun S. Singh, Bartosz Chmielowski, Noah C. Federman, Joseph G. Crompton, Brian E. Kadera, Lauren E. Wessel, Alexander B. Christ, Scott D. Nelson, Sarah M. Dry, Joanne B. Weidhaas, Michael L. Steinberg, Nicholas M. Bernthal, Fritz C. Eilber, Anusha Kalbasi, Vishruth K. Reddy

**Affiliations:** 1 Department of Radiation Oncology, University of California, Los Angeles; 2 Department of Radiation Oncology, University of Pennsylvania, Philadelphia; 3 Department of Surgery, University of Calgary, Calgary, Alberta, Canada; 4 Department of Medicine Statistics Core, University of California, Los Angeles; 5 Department of Radiation Oncology, University of California, Irvine; 6 Division of Hematology-Oncology, Department of Medicine, University of California, Los Angeles; 7 Department of Pediatrics, University of California, Los Angeles; 8 Division of Surgical Oncology, University of California, Los Angeles; 9 Department of Pathology and Laboratory Medicine, University of California, Los Angeles; 10 Department of Orthopedic Surgery, University of California, Los Angeles; 11 Department of Radiation Oncology, Stanford Cancer Institute, Stanford University School of Medicine, Stanford, California

## Abstract

**Importance:**

Standard preoperative radiotherapy (RT) for high-risk soft tissue sarcoma (STS) is delivered over 5 weeks, which can be a logistical challenge for patients.

**Objective:**

To evaluate the long-term toxic effects and clinical outcomes associated with a shorter 5-day, dose-equivalent preoperative RT regimen.

**Design, Setting, and Participants:**

Phase 2, single-group nonrandomized trial with an initial cohort (April 2016 to May 2018) and expansion cohort (October 2018 to May 2023) at a single academic center in the US. Participants were patients with histologically confirmed extremity or trunk STS recommended to undergo standard preoperative RT and surgery. Patients with planned neoadjuvant systemic therapy who were enrolled in the expansion group were excluded from this analysis. Analysis was conducted September 2024 to August 2025.

**Intervention:**

A total of 30 Gy in 5 fractions were delivered preoperatively.

**Main Outcomes and Measures:**

The primary end point was 2-year grade 2 or higher radiation toxic effects. Secondary end points included major wound complications (MWC), local failure, distant progression, and overall survival.

**Results:**

A total of 110 patients were treated with preoperative RT and surgery (42 patients [38%] were aged 65-79 years; 64 [58%] were male; 75 had tumors of the lower extremity [68%], and 64 patients [58%] had high-grade disease). The initial cohort accrued 50 patients who underwent surgery. The expansion cohort accrued 83 patients; 60 of 83 were treated without neoadjuvant chemotherapy and were included. Median (IQR) follow-up was 37.3 (20.1-60.6) months, including 64.2 (36.3-74.1) months for the initial cohort and 30.0 (13.5-40.2) months for the expansion cohort. At 2 years, 14 of 74 evaluable patients (18.9%) developed grade 2 or higher toxic effects (10 patients [25.0%] for the initial cohort and 4 patients [11.8%] for the expansion cohort). MWCs occurred in 33 of 110 patients (30.0%); (17 [34.0%] for the initial cohort and 16 [26.7%] for the expansion cohort). Time to wound closure exceeded 6 months for 15 patients (13.6%), including 12 of 29 patients (41.4%) who underwent local tissue advancement flaps. Two-year local control adjusting for competing risk of death was 92.4% (95% CI, 86.3%-96.5%). There were 3 (2.7%) bone fractures and 5 (4.5%) amputations.

**Conclusions and Relevance:**

This nonrandomized clinical trial of ultrahypofractionated preoperative RT identified durable local control with MWC and favorable late grade 2 or higher toxic effects rates. Randomized data are necessary to differentiate the safety profiles of various fractionation regimens, especially duration of wound healing.

**Trial Registration:**

ClinicalTrials.gov Identifier: NCT02701153

## Key Points

**Question:**

What safety and clinical outcomes are associated with a shorter 5-day preoperative radiotherapy regimen for extremity and truncal soft tissue sarcoma?

**Findings:**

In this nonrandomized phase 2 trial of 110 patients, median follow-up for the initial and expansion cohorts was 64.2 and 30.0 months, respectively. Major wound complications occurred in 33 of 110 patients (30.0%); at 2 years, 14 of 74 evaluable patients (18.9%) developed grade 2 or higher toxic effects. Two-year local control was 92.4% (95% CI, 86.3%-96.5%), and time to wound closure exceeded 6 months for 15 of 110 patients (13.6%).

**Meaning:**

These findings suggest 5-day preoperative radiotherapy was associated with favorable long-term clinical outcomes, but its potential impact on time-to-wound closure should be assessed in a randomized study.

## Teaser

This nonrandomized clinical trial evaluates the long-term toxic effects and clinical outcomes associated with a shorter 5-day, dose-equivalent preoperative radiation therapy (RT) regimen among patients with soft tissue sarcoma.

## Introduction

National guidelines recommend the addition of radiotherapy (RT) to surgery for patients with high-grade soft tissue sarcoma (STS), supported by randomized data demonstrating improvement in local control with the addition of RT.^1,2,3,4^ Preoperative RT may offer advantages compared with postoperative RT for patients, including smaller treatment volume and lower dose, which translates to a lower risk of late radiation-induced complications, such as edema, fibrosis, and joint stiffness.^5^ Despite these advantages, standard preoperative RT spans 5 weeks, which can be a logistical challenge to patients. This is especially true for patients with rare cancers such as sarcoma, who often travel long distances to receive care at tertiary cancer centers and are anxious to proceed with surgical resection.^6,7,8,9^

We conducted a prospective phase 2 trial to study an ultrahypofractionated 5-day preoperative RT regimen for STS of the extremities or trunk that delivers 30 Gy over 5 fractions,^10^ designed to mirror the biological effects of standard 5-week RT (equivalent dose [EQD2] of 50 Gy, using α/β = 4).^11,12^ In the 2-year outcomes of the original cohort, we reported favorable rates of major wound complications (MWCs) and late radiation-associated toxic effects. Since this report, other single-institution studies of 5-day RT have reported rates of MWCs ranging from 25% to 41%.^13,14,15^ In addition, 2 single-institution studies of moderately hypofractionated preoperative RT (42.75 Gy in 15 fractions) demonstrated acceptable rates of MWCs and radiation-associated toxic effects.^16,17^

However, there remain concerns about the long-term safety and disease control with hypofractionated preoperative RT for STS, and it is unclear whether ultrahypofractionated or moderately hypofractionated regimens provide a more suitable risk-benefit profile. Meanwhile, a recent consensus statement from international sarcoma experts at Connective Tissue Oncology Society recommended that hypofractionated preoperative RT regimens should not yet be standard of care outside of clinical trials.^18^

## Methods

### Study Design

This was a single-center, open-label, phase 2 nonrandomized clinical trial performed as part of a larger, ongoing trial. The protocol was approved by the University of California, Los Angeles institutional review board and appears in Supplement 1. Written, informed consent was obtained from all patients. This study followed the Transparent Reporting of Evaluations With Nonrandomized Designs (TREND) reporting guidelines.

### Participants

Eligible patients had histologically confirmed STS of the extremity or trunk and were recommended to have preoperative RT and surgery after multidisciplinary case review. There were no specific restrictions with regards to grade, size, or histology. All patients were aged 18 years or older with Eastern Cooperative Oncology Group performance statuses of 0 to 2. Exclusion criteria included evidence of distant metastases at time of enrollment, prior RT to the primary tumor, and active treatment of a second malignant neoplasm.

For the original cohort, an additional exclusion criterion was planned neoadjuvant or adjuvant chemotherapy. This was not applicable to the expansion cohort, which was opened to compare MWC rates between patients receiving preoperative RT and patients receiving neoadjuvant chemotherapy followed by preoperative RT. Patients in the chemotherapy group were excluded from this analysis. Concurrent chemotherapy was not allowed in either cohort.

### Procedures

Eligible patients were assigned to receive preoperative RT followed by surgery within 12 weeks. Computed tomography (CT) and/or magnetic resonance imaging (MRI) simulation was performed with custom immobilization. The gross tumor volume (GTV), clinical target volume (CTV), and planning target volume (PTV) were defined according to Radiation Therapy Oncology Group (RTOG) 0630.^18^ The GTV was defined by CT and T1-weighted MRI. The CTV was defined using a 3-cm longitudinal expansion and 1.5-cm radial expansion of the GTV, in addition to any suspicious edema seen on T2-weighted MRI. This was cropped out of uninvolved bone or nonadjacent muscle compartments. The PTV was generated using a 5-mm isotropic expansion of the CTV. The PTV was cropped 2 mm or more from the skin for superficial lesions unless there was skin involvement.

A dose of 6 Gy × 5 fractions (30 Gy) was delivered on consecutive weekdays to 95% or more of the PTV. Intensity-modulated radiotherapy (IMRT) was the preferred treatment modality, but selected cases were treated with 3-dimensional–conformal radiotherapy (3D-CRT) or electron planning techniques. RT plans were designed to meet dosimetric parameters outlined in eTables 1 and 2 in Supplement 2 for the original and expansion cohorts, respectively; PTV coverage was prioritized. All patients underwent daily image guidance except patients receiving electron RT. Surgical procedures were performed at UCLA by dedicated sarcoma surgeons (J.G.C., B.E.K., L.E.W., A.B.C., N.M.B., and F.C.E.). Pathology was interpreted at UCLA by dedicated sarcoma pathologists (S.D.N. and S.M.D.).

Patients were seen after the completion of RT and before surgery by the radiation oncologist and/or sarcoma surgeon. Patients were followed up clinically and radiographically by the radiation oncologist and/or sarcoma surgeon with CT or MRI of the primary site and CT of the chest every 6 months for the first 2 years and then annually.

### Outcome Measures

The primary end point was the 2-year rate of grade 2 or higher radiation morbidity (fibrosis, lymphedema, or joint stiffness). Patients who died or were lost to follow-up before 2 years of follow-up were excluded from the primary end point analysis. Fibrosis and joint stiffness were graded using RTOG or European Organisation for Research and Treatment of Cancer criteria. Lymphedema was graded using the Stern scale. This mirrored RTOG-0630.^19^ A sample size of 51 patients was chosen for the original cohort to allow for 80% power to identify an improvement of 20% compared with the historical preoperative RT group of the CAN-NCIC-SR2 study (17% vs 37%).^5^ This assumed approximately 20% of patients not being analyzable for the 2-year primary end point.

Secondary end points included acute (<90 days) adverse events using Common Terminology Criteria for Adverse Events (CTCAE) version 4.0, the rate of MWCs, freedom from local failure, freedom from distant metastasis, and overall survival (OS). MWCs were defined as requiring secondary operations for wound treatment (eg, debridement, flap procedure, or skin grafts), hospital readmission for wound care, invasive procedures required for wound care (eg, hematoma drainage or vacuum-assisted closure therapy) to an area of the wound measuring 2 or more cm, prolonged dressing changes (>6 weeks from wound breakdown), repeat surgery for revision of split-thickness skin graft, and applying wet dressings for less than 4 weeks. These were adapted from the randomized CAN-NCIC-SR2 trial.^5^ Local failure was defined as disease recurrence in or adjacent to the radiated field, while distant progression was defined as disease recurrence at a new metastatic site. Regional control was mentioned in the original protocol as a secondary end point but was not examined due to a lack of nodal progression events in either cohort.

### Statistical Analysis

Descriptive statistics summarized patient and treatment characteristics. Comparisons were made between the original and expansion cohorts using χ^2^ tests for categorical variables and *t* tests for continuous variables. Freedom from local failure and distant metastasis were calculated using the Aalen-Johansen method to adjust for the competing risk of death. OS was estimated using the Kaplan-Meier method. Local control was measured from the date of surgery. All other time-to-event end points were measured from the end of RT.

In unplanned exploratory analysis, we compared oncological outcomes between the original and expansion cohorts using log-rank testing to evaluate for differences between the 2 cohorts. Multiple logistic regression was used to evaluate the association between grade 2 or higher radiation-associated toxic effects and tumor size (continuous variable), involvement of the adductor compartment (yes or no), involvement of the elbow or knees (yes or no), and RT technique (IMRT vs 3D-CRT or electrons). Cox regression was used to evaluate the association between time to local failure and grade (categorical variable), tumor size (continuous variable), prior local resection (yes or no), and positive surgical margin (yes or no). Multiple logistic regression was used to evaluate the association between MWCs and initial surgical closure method (vascularized flap, local tissue advancement, or primary closure), involvement of the adductor compartment (yes or no), involvement of the elbow or knees (yes or no), diabetes (yes or no), and smoking status (current smoker vs never or former smoker). χ^2^ Tests were used to compare the number of open wounds at 6 months across the 3 closure methods (vascularized flap, local tissue advancement, or primary closure). No corrections for multiple comparisons or the competing risk of death were applied due to the exploratory nature of the analysis.

In all analyses, *P* < .05 was considered statistically significant. Analyses were performed using R version 4.4.0 (R Project for Statistical Computing) and GraphPad Prism, version 10.2.2 for Mac (GraphPad Software) between September 2024 and August 2025.

## Results

### Patient Characteristics

One hundred and ten patients were treated with preoperative RT and surgery. A total of 42 patients (38%) were aged 65 to 79 years, and 64 (58%) were male. A total of 75 had tumors of the lower extremity (68%), 16 of the upper extremity (15%), and 19 of the trunk (17%); 7 had tumors that were low grade (6%), 31 were intermediate grade (28%), and 64 were high grade (58%); and 26 had tumor diameter 5 cm or less (24%), 50 were larger than 5 cm but 10 cm or less (45%), and 34 were larger than 10 cm (31%) (Table).

**Table.** Tumor and Patient Characteristics of the Original, Expansion, and Overall Cohorts

Characteristic	No. (%)			*P* value^a^
	Overall (N = 110)	Original cohort (n = 50)	Expansion cohort (n = 60)	
Age				
<50 y	31 (28)	14 (28)	17 (28)	.64
50-64 y	29 (26)	11 (22)	18 (30)	
65-79 y	42 (38)	20 (40)	22 (37)	
>79 y	8 (7)	5 (10)	3 (5)	
Sex				
Female	46 (42)	23 (46)	23 (38)	.42
Male	64 (58)	27 (54)	37 (62)	
ECOG status				
0	80 (73)	39 (78)	41 (68)	.02
1	24 (22)	6 (12)	18 (30)	
2	6 (5)	5 (10)	1 (2)	
Smoking history				
Yes	30 (27)	12 (24)	18 (30)	.48
No	80 (73)	38 (76)	42 (70)	
Diabetes history				
Yes	11 (10)	4 (8)	7 (12)	.52
No	99 (90)	46 (92)	53 (88)	
Histology				
Undifferentiated^b^	49 (45)	24 (48)	25 (42)	.29
Myxofibrosarcoma	13 (12)	8 (16)	5 (8)	
Myxoid liposarcoma	22 (20)	11 (22)	11 (18)	
Angiosarcoma	2 (2)	2 (4)	0	
Liposarcoma	5 (5)	2 (4)	3 (5)	
MPNST	2 (2)	1 (2)	1 (2)	
Pleomorphic sarcoma	4 (4)	1 (2)	3 (5)	
Leiomyosarcoma	4 (4)	1 (2)	3 (5)	
Chondrosarcoma	2 (2)	0	2 (3)	
Spindle cell sarcoma	1 (1)	0	1 (2)	
Synovial sarcoma	2 (2)	0	2 (3)	
Other	4 (4)	0	4 (7)	
Primary site				
Upper extremity	16 (15)	9 (18)	7 (12)	.52
Lower extremity	75 (68)	34 (68)	41 (68)	
Trunk	19 (17)	7 (14)	12 (20)	
Size				
≤5 cm	26 (24)	13 (26)	13 (22)	.36
>5 cm and ≤10 cm	50 (45)	25 (50)	25 (42)	
>10 cm	34 (31)	12 (24)	22 (37)	
Grade				
Low	7 (6)	1 (2)	6 (10)	.01
Intermediate	31 (28)	19 (38)	12 (20)	
High	64 (58)	30 (60)	34 (57)	
Not specified	8 (7)	0	8 (13)	
Time between RT and surgery, median (IQR), d	26 (19-33)	27.5 (21-32)	24.5 (18-33)	.60
RT planning technique				
3D-CRT or electrons	13 (12)	12 (24)	1 (2)	.001
IMRT	97 (88)	38 (76)	59 (98)	

Abbreviations: 3D-CRT, 3-dimensional conformal radiation therapy; ECOG, Eastern Cooperative Oncology Group; IMRT, intensity-modulated radiotherapy; MPNST, malignant peripheral nerve sheath tumor; RT, radiotherapy.

^a^ Comparisons between the original and expansion cohorts were made using χ^2^ tests for categorical variables and *t* tests for continuous variables.

^b^ Includes undifferentiated pleomorphic sarcoma and undifferentiated spindle cell sarcoma.

**Figure 1.**
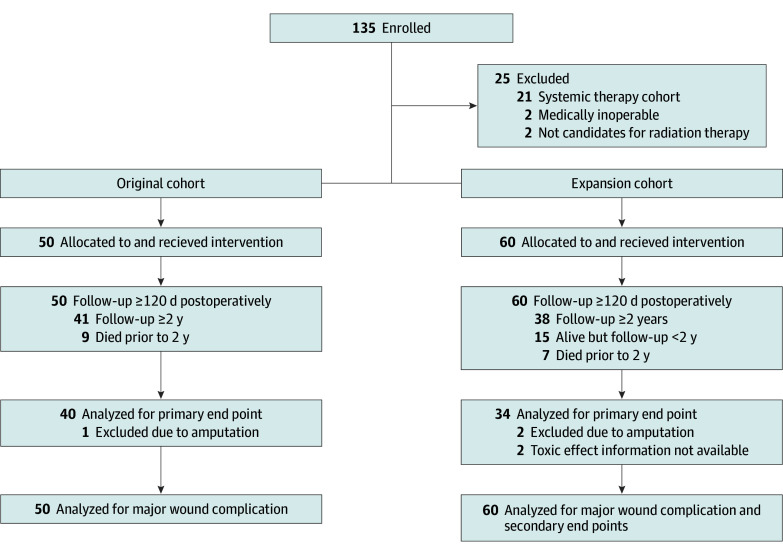
Diagram of the Original and Expansion Cohorts

Between April 2016 and May 2018, 52 patients with localized high-risk STS of the extremity or trunk were enrolled in the original cohort. Fifty ultimately underwent preoperative RT and surgery (Figure 1). Between October 2018 and February 2023, 83 patients enrolled in our expansion cohort; 60 of 83 underwent preoperative RT without preoperative systemic therapy and were included in the current analysis. Median (IQR) follow-up was 64.2 (36.3-74.1) months for the initial cohort and 30.0 (13.5-40.2) months for the expansion cohort. The initial and expansion cohorts had similar characteristics in terms of histology, primary site, tumor size, time between RT and surgery, age, sex, smoking history, and diabetes. There was more frequent use of IMRT in the expansion cohort (98% vs 76%; χ^2^_2_ = 25.8; *P* = .002).

The median (IQR) time between completion of RT and surgery was 26 (19-33) days. Most patients had R0 resections (91 of 110 patients [82.7%]). Nineteen patients (17.3%) had R1 resections; 9 subsequently underwent re-excision to attempt an R0 resection. This decision was based on whether an R0 resection was thought to be feasible and if reresection would not be expected to cause significant morbidity. Seventy-six patients (69.1%) underwent primary closure, 29 (26.4%) received local tissue advancement flaps, and 5 (4.5%) received vascularized tissue grafts.

### Radiation-Associated Toxic Effects

After a minimum of 2 years of follow-up, 14 of 74 evaluable patients (18.9%) experienced grade 2 or higher radiation-associated toxic effects (12.2% developed fibrosis, 12.2% joint stiffness, and 4.1% lymphedema). This included 1 case (1.4%) of grade 3 joint stiffness and 1 case (1.4%) of grade 3 radiation fibrosis. Grade 2 or higher radiation-associated toxic effects were significantly associated with tumor size (odds ratio [OR], 1.17; 95% CI, 1.06-1.32; *P* = .004), but not RT technique, involvement of the adductor compartment, or involvement of the elbows or knees (eTable 3 in Supplement 2).

In the initial cohort, 10 of 40 evaluable patients (25.0%) had grade 2 or higher radiation-associated toxic effects (17.5% developed fibrosis, 17.5% joint stiffness, and 5% lymphedema). In the expansion cohort, 4 of 34 evaluable patients (11.8%) had grade 2 or higher radiation-associated toxic effects (5.9% developed fibrosis, 5.9% joint stiffness, and 2.9% lymphedema). Grade 1 radiation-associated toxic effects rates were 19 of 40 (47.5%) in the original cohort and 34 of 60 (56.7%) in the expansion cohort. Grade 3 radiation-associated toxic effects rates were 2 of 40 (5%) in the original cohort and 0 of 34 in the expansion cohort.

There were 4 of 110 (3.6%) grade 3 bony complications with 3 (2.7%) fractures. One fracture occurred 2 months postoperatively in a patient whose tumor eroded into the humerus at diagnosis and was not attributed to radiation. There was 1 case of a radiation-associated fracture of the humerus (14 months postoperatively), 1 case of radiation necrosis of the humerus (5 years postoperatively), and 1 case of a radiation-associated fracture of the ilium (4 months postoperatively). All radiation-associated fractures occurred in the setting of periosteal stripping and did not receive prophylactic surgical stabilization. All met dose constraints. The 3 cases involving the humerus were treated with surgical fixation, while the case involving the ilium was treated nonoperatively. There were no cases of radiation-associated radiculopathy.

Acute grade 2 toxic effects occurred in 9 of 110 patients (8.2%). Acute grade 2 toxic effects consisted of radiation dermatitis in 5 patients, pain in 2, limb edema in 1, and nausea in 1. There were no grade 3 or higher acute toxic effects.

### Wound Complications

Twenty-four patients (21.8%) experienced grade 3 or higher CTCAE wound complications, including 12 of 50 patients (24.0%) in the initial cohort and 12 of 60 (20.0%) in the expansion cohort. MWCs occurred in 33 patients (30.0%), including 16 of 50 (32.0%) in the initial cohort and 17 of 60 (28.3%) in the expansion cohort. Nineteen patients (17.3%) experienced wound dehiscence a median (IQR) of 43 (38-68) days after surgery, 13 (11.8%) experienced soft tissue infections, and 11 (10.0%) experienced soft tissue radiation necrosis.

Rates of MWCs were 18.4% (14 of 76 patients) for primary closure, 62.1% for local tissue advancement flaps (18 of 29 patients), and 20.0% (1 of 5 patients) for vascularized tissue grafts. Patients who underwent local tissue advancement flaps with their initial surgery were more likely to have MWCs (OR, 14.33; 95% CI. 4.81-48.88; *P* < .001) compared with primary closure (eTable 4 in Supplement 2). Diabetes was associated with MWCs (OR, 6.09; 95% CI, 1.39-27.11; *P* = .02). There was no association between MWC rates and involvement of the adductor compartment, involvement of the elbow or knees, or smoking history (eTable 4 in Supplement 2).

At time of analysis, 31 of 33 patients with MWCs achieved wound closure a median (IQR) of 172 (126-260) days postoperatively. Of the remaining 2 patients, 1 died before wound closure due to unrelated causes, while the other achieved wound closure after the time of this analysis. Among the 33 patients with an MWC, 15 had an open wound 180 days postoperatively (13.6% of all patients). Nine sites were lower extremity, 2 were upper extremity, and 4 were truncal; 5 required irrigation and debridement. Notably, the rate of open wounds at 180 days was 41.4% (12 of 29) for local tissue advancement flaps vs 3.9% (3 of 76) for primary closure and 0 of 5 for vascularized tissue grafts (χ^2^_2_ = 25.8; *P* < .001).

Thirty-four patients (30.9%) required a second surgery a median (IQR) of 77 (37-138) days from the index surgery. Fourteen were re-excisions, 16 were irrigation and debridement, 2 were amputations, and 2 were open reduction and internal fixation. Subsequently, there were 3 additional amputations and 1 open reduction and internal fixation. The overall rate of limb salvage was 95.5% (105 of 110). There were 5 total amputations, all performed due to disease progression, a median (IQR) of 150 (140-230) days postoperatively, including 1 in the initial cohort and 4 in the expansion cohort. Three were below the knee, 1 was above the knee, and 1 involved hip disarticulation.

### Oncologic Outcomes

**Figure 2.**
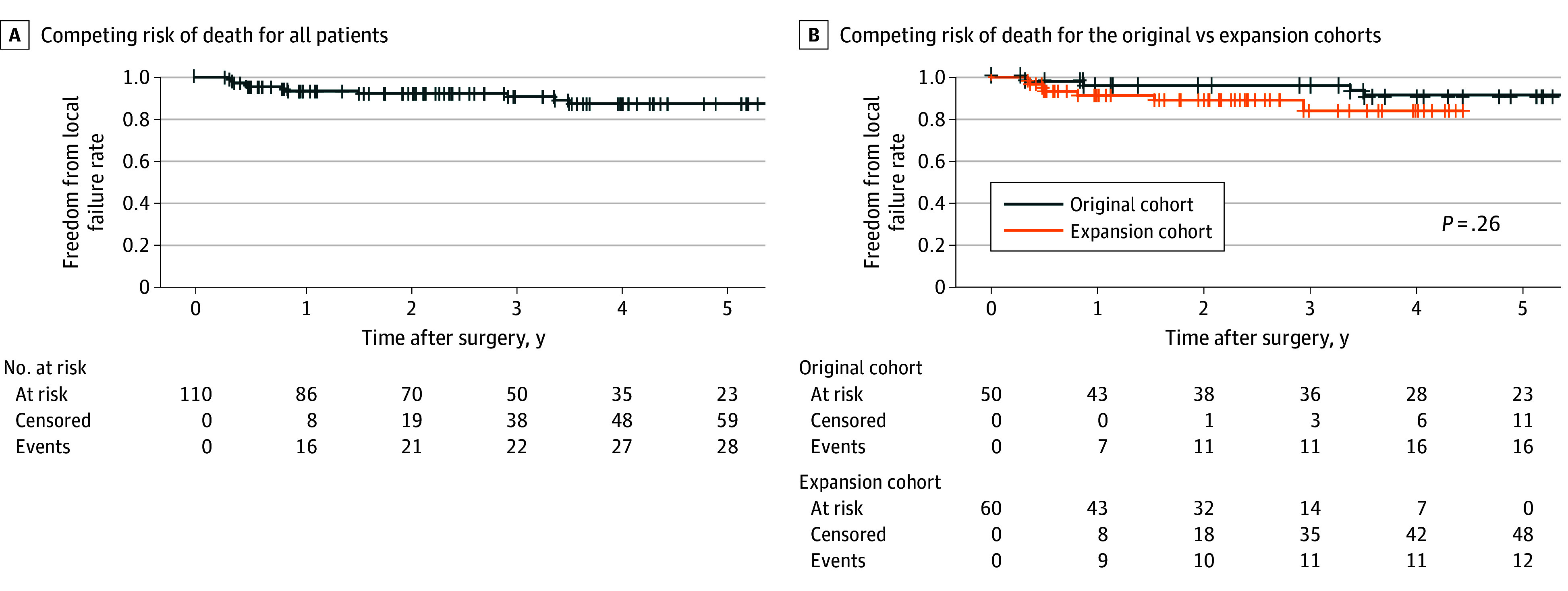
Freedom From Local Failure A, Freedom from local failure using the Aalen-Johansen method to adjust for the competing risk of death for all patients. B, Freedom from local failure using the Aalen-Johansen method to adjust for the competing risk of death for the original vs expansion cohorts. Comparisons were made using log-rank testing.

The 2-year freedom from local failure rate adjusting for competing risk of death was 92.4% (95% CI, 86.3%-96.5%) (Figure 2A). There was no significant difference between the original and expansion cohorts (Figure 2B). Local failures were more likely after resections with microscopically positive margins (hazard ratio, 4.81; 95% CI, 1.07-21.25; *P* = .03). They were not associated with tumor size, grade, or history of prior resection (eTable 5 in Supplement 2).

**Figure 3.**
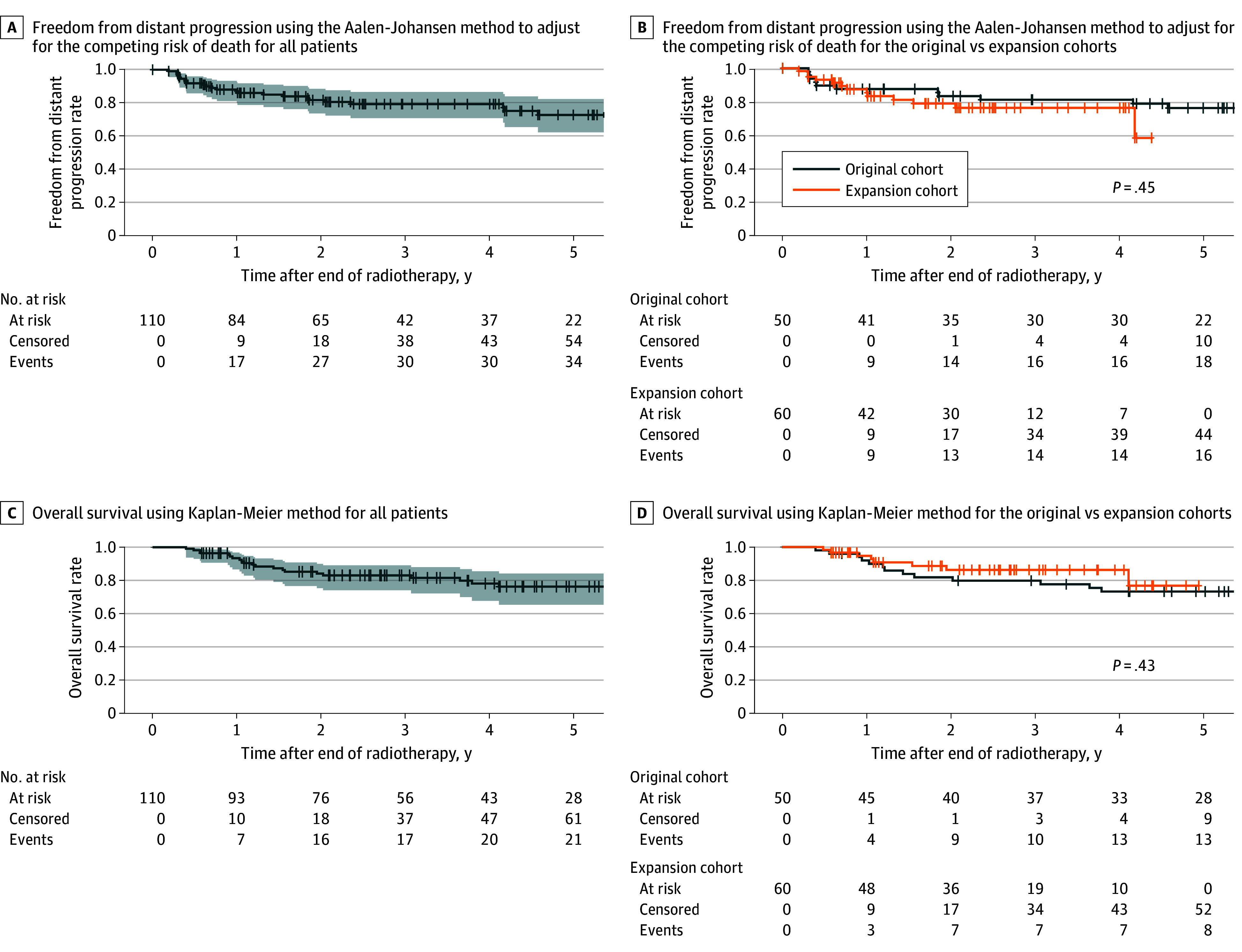
Freedom From Distant Progression and Overall Survival A, Freedom from distant progression using the Aalen-Johansen method to adjust for the competing risk of death for all patients; 95% CIs are shown in shaded area. B, Freedom from distant progression using the Aalen-Johansen method to adjust for the competing risk of death for the original vs expansion cohorts. Comparisons were made using log-rank testing. C, Overall survival using Kaplan-Meier method for all patients; 95% CIs are shown in shaded area. D, Overall survival using Kaplan-Meier method for the original vs expansion cohorts. Comparisons were made using log-rank testing.

The 2-year freedom from distant progression rate adjusting for the competing risk of death was 81.6% (95% CI, 73.5%-88.4%) (Figure 3A). There was no significant difference between the original and expansion cohorts (Figure 3B). The 2-year OS rate was 82.9% (95% CI, 73.9%-89.0%) (Figure 3C). There was no significant difference between the original and expansion cohorts (Figure 3D).

## Discussion

STS are rare tumors, composing less than 1% of diagnosed malignant neoplasms annually. Prior studies indicated that treatment at high-volume sarcoma centers is associated with improved outcomes.^20,21,22^ In addition to serving as a barrier to treatment at these centers, the standard 5-week course of radiation poses a logistical challenge for patients, often contributing to the underutilization of RT even when indicated.^23,24^ Hypofractionated RT regimens not only align more closely with patient preferences,^25^ but also reduce travel burdens and enhance access to care at high-volume centers. Shorter RT schedules continue to be an active area of investigation for various malignant neoplasms, including breast and prostate cancers.^26,27^

There is emerging evidence for hypofractionated preoperative RT regimens for the treatment of STS of the extremities and trunk. One of the largest studies to date is a single-institution series of 120 patients treated with moderately hypofractionated preoperative RT, demonstrating excellent local control (93% at 30 months), acceptable MWC rate (31%), and low rate of late toxic effects.^16^ Several studies, including our early report of the initial cohort described here, have demonstrated favorable local control and MWC rates for ultrahypofractionated 5-day preoperative RT regimens; however, these small single-institution series are limited in scope.^10,13,14,15,16,17^ Concerns about toxic effects, including fibrosis, fracture risk, radiculopathy, MWCs, persistent open wounds, and amputations, have been raised, leading to expert consensus that hypofractionated preoperative RT regimens should not yet be considered standard-of-care.^18^

In our study, we demonstrate an acceptable rate of 2-year grade 2 or higher radiation-associated toxic effects (18.9%), which remains favorable compared with the CAN-NCIC-SR2 study against which our study was powered (18.9% vs 37%), although it is higher than recent multi-institutional phase 2 data using modern image-guided 5-week preoperative RT (18.9% vs 10.5%).^19^ We found that the expansion cohort of this trial had a lower 2-year grade 2 or higher radiation-associated toxic effects rate than the original cohort (11.8% vs 25.0%), which may be partly related to improved experience with ultrahypofractionated RT in this setting or increased IMRT use. It is important to note that these events are graded qualitatively, necessitating cautious interpretation of these differences.

The increased rate of MWCs is the primary downside of any preoperative sequencing of RT in the treatment of STS, hypofractionated or otherwise.^5^ The MWC rate in our study (30%) aligns with the results from previous prospective and retrospective studies that used conventional 5-week preoperative RT (22%-37%).^5,11,19^ We reported that at 6 months, 13.6% of patients still had an open wound. This appears to be related to the specific surgical closure technique that was used. Following local tissue advancement flaps, 41% of patients (12 of 29) had an open wound at 6 months vs 4% (3 of 81) who underwent primary closure or vascularized tissue flaps. The latter group of patients compares favorably with a recently published moderately hypofractionated series showing an overall open wound rate of 6% at 6 months.^17^ This highlights the potential need for alternative closure methods in cases where primary closure is challenging, potentially involving more frequent collaboration with plastic surgery specialists to achieve adequate vascularized soft tissue reconstruction.

One of the significant concerns associated with hypofractionated regimens is the potential for late radiation-induced toxic effects, particularly due to the low alpha and beta ratio of late-responding tissues, such as bone and peripheral nerves, which can result in an elevated risk of complications like fractures and radiculopathy. In our study, we found no incidence of radiculopathy, which is consistent with our radiation dose being below the reported tolerance for peripheral nerves. Additionally, our fracture rate was 2.7%, which is higher than the 1.4% fracture rate observed in a recent series of 700 patients for whom bone avoidance objectives were implemented.^28^ We believe that our patient cohort would have benefited from more frequent prophylactic surgical stabilization. Our practice was to recommend it in cases where we deemed a higher risk for fractures after multidisciplinary discussion of patient comorbidities and the nature of the resection (such as the need for periosteal stripping). Finally, our study reported a 4.5% amputation rate. Given our role as a tertiary sarcoma referral center, we often treat patients for whom amputation was recommended at an outside facility but who are motivated for limb salvage. We offer them limb-sparing radiation therapy with the caveat that amputation may still be the eventual outcome. This likely affects our reported limb salvage rate.

With regards to long-term oncological outcomes, we reported outcomes that were broadly consistent with contemporary series of preoperative RT. The investigators of RTOG-0630 reported that their 2-year freedom from local recurrence rate was 94.0% vs 92.4% in our study, their 2-year freedom from distant recurrence rate was 62.7% vs 81.6% in our study, and their 2-year overall survival rate was 80.6% vs 82.9% in our study.^19^ Furthermore, the outcomes of our initial and expansion cohorts were comparable for all oncological outcomes, highlighting the consistency of our results over a 7-year period.

### Limitations

There are limitations of this study. First, this is a single-institution experience. Our results may not necessarily be generalizable to lower volume centers with less experience treating STS, both from an RT and surgical standpoint. Second, this is a nonrandomized, single-arm trial. Conducting large, randomized studies remains an ongoing challenging for rare malignant neoplasms such as STS. Third, physician-reported toxic effects grading involves qualitative interpretations and can vary between physicians. We noticed differences in the grade 1 vs grade 2 toxic effects rates between the original and expansion cohorts that may be due to differences in physician grading as the sarcoma team lost and gained new investigators. Additionally, these patients were not treated with chemotherapy or immunotherapy, which is being examined in a separate cohort.

## Conclusions

In this clinical trial study of ultrahypofractionated 5-day preoperative RT in extremity and trunk STS, we reported clinical outcomes comparable with those observed with conventionally fractionated preoperative RT. Although MWC rates were consistent with those observed after standard fractionation, we observed prolonged time to wound closure in a subset of patients warranting further evaluation of this understudied variable. Future randomized study comparing 5-day preoperative RT with conventional or moderately hypofractionated regimens should carefully weigh any potential increased toxic effects risk against the benefits of a more accessible regimen.

## Notes

Supplement 1. Trial Protocol

Supplement 2. eTable 1. Dosimetric Parameters for Radiation Planning for the Original Cohort eTable 2. Dosimetric Parameters for Radiation Planning for the Expansion Cohort eTable 3. Multiple Logistic Regression Analysis for Associations With Experiencing Grade 2 or Higher Radiation-Associated Toxicities eTable 4. Multiple Logistic Regression Analysis for Associations With Experiencing a Major Wound Complication eTable 5. Cox Regression Analysis for Associations With Experiencing Local Failure

Supplement 3. Data Sharing Statement
