# Supplementary material for: Five-Day Preoperative Radiation Therapy for Patients With High-Risk Soft Tissue Sarcoma: A Nonrandomized Clinical Trial
Source: JAMA Netw Open. 2025 Dec 17;8(12):e2550195. doi: 10.1001/jamanetworkopen.2025.50195 (PMC12712729; doi:10.1001/jamanetworkopen.2025.50195)
Supplement: Supplement 1. — Trial Protocol [file jamanetwopen-e2550195-s001.pdf]

Phase II Study of 5-Day Hypofractionated Preoperative Radiation Therapy for Soft Tissue Sarcomas: Expansion Cohort

Principal Investigator:
Vishruth Reddy, M.D.
UCLA Radiation Oncology/UCLA Jonsson Comprehensive Cancer Center

Co-Investigators:
Frederick (Fritz) Eilber, M.D.
UCLA Surgical Oncology

Nicholas Bernthal, M.D.
UCLA Orthopedic Surgery

Arun Singh, M.D.
UCLA Hematology/Oncology

Bartosz Chmielowski, M.D. Ph.D.
UCLA Hematology/Oncology

Noah Federman, M.D.
UCLA Pediatric Oncology

John Nikitas, MD
UCLA Radiation Oncology

Nicholas J. Jackson, M.P.H, Ph.D.
UCLA Department of Medicine Statistics Core
Division of General Internal Medicine and Health Services Research

Anusha Kalbasi, M.D.
Radiation Oncology/Stanford Cancer Institute

Study Coordinator:
Jackie Hernandez
UCLA Radiation Oncology/UCLA Jonsson Comprehensive Cancer Center

Study Centers:
UCLA Jonsson Comprehensive Cancer Center
Department of Radiation Oncology
200 UCLA Medical Plaza, Suite B265
Los Angeles, CA 90095

Stanford Cancer Institute
Department of Radiation Oncology
Lorry Lokey Building/SIM 1
265 Campus Drive, Ste G2103
Stanford, CA 94305

INDEX

PROTOCOL SYNOPSIS

SCHEMA

1.0 OBJECTIVES

2.0 BACKGROUND

3.0 PATIENT SELECTION

4.0 REGISTRATION PROCESS

5.0 TREATMENT PLAN

6.0 PHARMACEUTICAL INFORMATION

7.0 ADVERSE EVENTS: LIST AND REPORTING REQUIREMENTS

8.0 STUDY CALENDAR

9.0 DATA REPORTING/REGULATORY CONSIDERATIONS

10.0 STATISTICAL CONSIDERATIONS

11.0 REFERENCES

PROTOCOL SYNOPSIS:

Radiation therapy in either the pre- or post-operative setting is a standard-of-care treatment option for patients with soft tissue sarcomas based on randomized level 1 evidence(*1*). Pre-operative radiation treatment is favored at many institutions over post-operative treatment, most notably because it allows the delivery of a lower dose of radiation to a smaller volume(*2*). The standard pre-operative radiation dose is 45-50 Gy delivered in 25 treatment sessions over 5 weeks.

The 5-week course of radiotherapy can be challenging for sarcoma patients, many of whom travel great distances to have their care at specialized tertiary sarcoma centers. Logistical hurdles prevent patients from relocating to these tertiary centers for the duration of their radiation treatment. We proposed a shorter, condensed form of hypofractionated preoperative radiation therapy administered over 5 days.

More condensed forms of radiation therapy have been adopted in the treatment of several malignancies as radiation oncologists can more easily spare normal tissues with modern radiation techniques and image guidance(*3, 4*). There is also a biological rationale for this approach in sarcoma, a tumor that is less sensitive to smaller RT fraction sizes (lower α/β ratio; (*5*)). The purpose of this study is to investigate the toxicity and disease control outcomes of a 5-day radiation regimen that is biologically equivalent to the standard 45-50 Gy regimen given over 5 weeks (25 treatments).

We recently completed an initial cohort of this study (n=52), which was limited to patients receiving neoadjuvant radiation therapy alone. Initial findings of this study have been reported after over 2.5 years of follow-up, and indicate acceptable rates of short- and medium-term complications, with promising rates of local control, both on par with rates observed using conventional 5-week preoperative radiation(*6*).

In our initial cohort, we excluded patients receiving neoadjuvant chemotherapy, which is given in patients with highest-risk disease. Currently, patients receiving neoadjuvant chemotherapy receive conventional 5-week preoperative radiation therapy, or in some cases, an abbreviated 8-day course of 28 Gy(*7*). Now, we propose an expansion cohort to examine our novel 5-day regimen in patients receiving either neoadjuvant RT alone or neoadjuvant RT following neoadjuvant chemotherapy. The goal of this expansion cohort is to compare toxicity outcomes in patients receiving neoadjuvant chemotherapy with patients receiving radiotherapy alone. Should the rate of toxicity be similar in each cohort, then the 5-day approach can become incorporated as a standard-of-care for all patients regardless of the use of neoadjuvant chemotherapy.

PRIMARY OBJECTIVE (s):

- In patients undergoing preoperative radiotherapy for extremity or trunk soft tissue sarcoma, compare the major wound complication rate in patients treated with neoadjuvant chemotherapy versus patients receiving neoadjuvant radiotherapy alone.

SECONDARY OBJECTIVE (s):

- Evaluate the 2 year rate of grade ≥ 2 radiation morbidity (subcutaneous fibrosis, joint stiffness, or edema) with a 5 fraction radiation regimen that is biologically equivalent to a standard 45-50 Gy regimen given in 25 fractions.
- Evaluate local control, regional control, distant metastasis, progression free survival, and overall survival.
- Evaluate the functional outcomes as assessed using the musculoskeletal tumor rating scale (MSTS) and the Toronto Extremity Salvage Score (TESS),

EXPLORATORY OBJECTIVES:

- Investigate the association of germ-line mutations that impact cancer predisposition.
- Investigate the role of germ-line mutations in predicting cancer outcome, toxicity and response to therapy.
- Evaluate pre-treatment, post-radiotherapy and, if applicable, metastatic or relapse tissue specimens for characteristics including DNA alterations, gene expression, protein expression, immunophenotype and intratumoral heterogeneity that may be predictive, prognostic or explain resistance.

STUDY DESIGN:

Single arm, non-randomized, phase II trial of preoperative hypofractionated radiation for extremity/trunk soft tissue sarcomas

STRATIFICATION:

Patients will be stratified into two cohorts according to the previous administration of neoadjuvant chemotherapy.

Cohort A: neoadjuvant radiotherapy alone
Cohort B: neoadjuvant chemotherapy and radiotherapy

NUMBER OF PATIENTS
ORIGINAL COHORT: 51 patients

EXPANSION COHORT:

Cohort B: 41 patients (minimum)
Cohort A: 123 patients (minimum)

The study will be considered having achieved minimal accrual when both cohorts have reached their minimum enrollment. The total enrollment goal for the expansion cohort is 205 patients to account for an estimated 20% of patients having a screening failure or dropping out from the trial.

ELIGIBILITY CRITERIA:

- Histologically confirmed soft tissue sarcoma of the extremity/trunk.
- Planning to undergo standard preoperative radiotherapy
- Resectable primary lesion (patients with pre-existing metastasis will be included if the primary lesion will be resected)
- Age ≥ 12
- KPS ≥ 70 or ECOG 0-2
- If a woman is of childbearing potential, a negative serum or urine pregnancy test must be documented.

EXCLUSION CRITERIA:

- Active treatment of a separate malignancy
- History of prior irradiation to the area to be treated

INTERVENTION AND MODE OF DELIVERY:

Hypofractionated radiation will be delivered in the pre-operative setting to patients with soft tissue sarcoma with external beam radiation therapy. Patients in cohort A will receive 6.0 Gy x 5 fractions delivered daily in 5 fractions. Patients in cohort B will receive 5.0 Gy x 5 fractions delivered daily in 5 fractions. Patients will not be treated on weekends or holidays as is standard practice for radiation treatments.

DURATION OF INTERVENTION AND EVALUATION:

Patients will be seen either by the radiation oncologist or the surgeon within the first 3 months after completion of pre-operative radiation therapy. For patients who live a distance far enough away from UCLA where travel would be challenging for the patient a phone follow-up will be considered acceptable. After this, follow-up will be performed every 6 months after the time of surgery for the 5 years after treatment. The patients will be followed both clinically and radiographically after treatment. Follow up phone or telemedicine visits will be allowed for some individuals where travel back to UCLA is challenging for the patient (i.e patients on this study may be enrolled who live in other states, such as Nevada). Surveillance imaging of the primary site by CT or MRI and of the chest by CT will be performed every 6 months for the first 2 years and then once at the end of year 3. Additional scans may be obtained at the investigators' discretion.

STATISTICAL METHODS:

1. Analysis of primary end point (major wound complication event).
1.1 The comparison of major wound complication rates, defined as number of patients with major wound complication events over total number of patients, in cohorts A and B will be assessed using one- sided two-sample proportion test.

2. Analysis of secondary endpoints
2.1 Interim Reports for the Primary Endpoint grade ≥ 2 radiation morbidity (subcutaneous tissue fibrosis, joint stiffness, or edema) at 2 years. Interim reports will be prepared yearly until the results of the study are published. In general, the interim reports will contain information about patient accrual rate with projected completion dates, status of QA review and compliance rate of treatment per protocol, and the frequencies and severity of toxicity.
2.2 Analysis of efficacy with 5 secondary endpoints (local control, regional control, distant control, progression-free survival, and overall survival)
2.2.1 Kaplan-Meier analysis will be carried out and used to estimate the local control, regional control, distant control, progression-free survival, and overall survival rates.

3. Analysis of exploratory endpoints
3.1 The association between these 5 secondary endpoints and potential predictors (tumor size at diagnosis, tumor grade, tumor depth, tumor immune infiltration, patient age, patient sex) will be assessed by Cox proportional hazards model.
3.2 The association between germline mutations and response, toxicity and cancer outcome will be investigated using logistic regression model.
3.3 The impact of radiation on local and systemic immune responses will be evaluated using tumor and blood specimens, and these immunologic changes will be related back to patient outcomes.

SAMPLE SIZE JUSTIFICATION:
The original study was designed to test for a 20% absolute improvement in the rate of grade ≥ 2 radiation morbidity (subcutaneous tissue fibrosis, joint stiffness, or edema at 2 years (± 3 months) from 37% in the preoperative RT arm of the CAN-NCIC-SR2 study to 17% which requires 41 patients per cohort with 5% type I error and 80% statistical power. Allowing for 20% of patients being ineligible or not analyzable at 2 years, the total sample size needed is 51 patients. **The study met this initial endpoint(*6*).**

The expansion cohort of this study aims to evaluate non-inferiority of major wound complication rate in cohort B (neoadjuvant chemotherapy and radiotherapy) versus patients in cohort A (radiotherapy alone). One-sided two-sample proportion test will be carried out to assess the non-inferiority of major wound complication rate in cohort B compared to cohort A. According to a recent study on rates of wound complications in patients receiving preoperative RT(*8*), we assume the wound complication rate in cohort A to be 21% (79% success rate). We also assume that for every patient enrolled in cohort B, at least three patients will be enrolled in cohort A. With significance level 0.1 and power 80%, the enrollment of at least 41 patients in cohort B will enable us to detect a 15% increase in the wound complication rate of cohort B, which would translate to a 36%

wound complication rate (64% success rate)(*9*). A wound complication rate of 36% has previously been considered acceptable for patients receiving standard preoperative radiotherapy and surgery(2). We set the minimum enrollment number to 123 for cohort A and 41 for cohort B. To account for a 20% rate of potential screening failures or participant dropouts, the total enrollment goal for the expansion cohort is 205 patients.

FUNDING, REGULATORY, AND FEASIBILITY ISSUES:

UCLA Dept. of Radiation Oncology has the capability, equipment, and expertise to perform this external beam radiation therapy treatment.

PATIENT ACCEPTABILTY/ETHICS AND CONSENT ISSUES:

Only patients able to give informed consent will be eligible for the trial.

SCHEMA

Diagnosis of soft tissue sarcoma of the extremity/trunk

↓

Plan for preoperative radiation treatment

↓

5 fractions of radiation (5.0 or 6.0 Gy x 5) delivered daily

↓

Surgery within 12 weeks post-radiation

↓

Follow-up

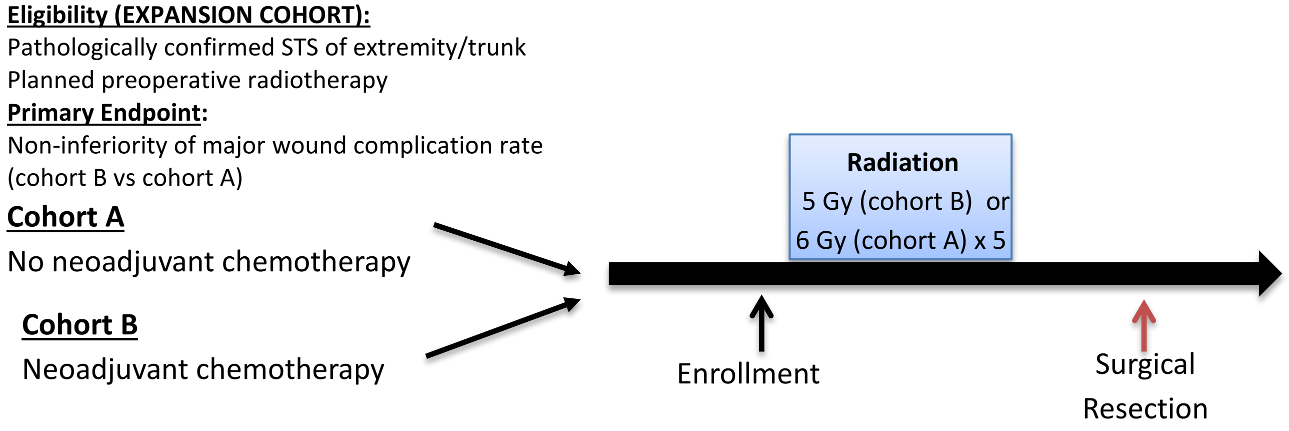

1.0 OBJECTIVES

1.1 Primary objectives

1.1.1 Primary endpoint:
Rate of major wound complications per the criteria set forth in Appendix

1.2 Secondary objectives

1.2.1 Secondary endpoints:
Grade ≥ 2 radiation morbidity (subcutaneous tissue fibrosis, joint stiffness, or edema) at 2 years

Evaluate local control, regional control, distant metastasis, progression free survival, and overall survival

Evaluate the functional outcomes as assessed using the musculoskeletal tumor rating scale (MSTS) and the Toronto Extremity Salvage Score (TESS)

1.3 Exploratory objectives

Collect germ-line DNA and nucleic acids from cancer patients to further investigate the association and identify new germ-line mutations that impact cancer predisposition

Investigate the role of germ-line mutations in predicting cancer outcome, toxicity, and response to therapy

Evaluate pre-treatment, post-radiotherapy and, if applicable, metastatic or relapse tissue specimens for characteristics including DNA alterations, gene expression, protein expression, immunophenotype and Intratumoral heterogeneity that may be predictive, prognostic or explain resistance.

2.0 BACKGROUND

Randomized data demonstrates that radiation therapy improves local control after surgical resection in extremity/trunk soft tissue sarcomas(*1*). Some advantages have been observed however with the administration of pre-operative compared with post-operative radiation. This includes a reduction in late term lymphedema, fibrosis and joint stiffness but comes at the cost of increased acute wound complications. Given that wound complications are temporary whereas lymphedema, fibrosis, and joint stiffness are permanent injuries many groups prefer to deliver pre-operative radiation.

The standard dose for pre-operative radiation therapy is 45-50 Gy delivered daily over the course of about 25 treatments. This is an effective treatment however it is inconvenient for patients to come daily for treatment over 5 weeks. This means that many patients receive their radiation therapy in the community under the care of a physician who may see only a few sarcomas each year whereas a radiation oncologist at a specialized sarcoma center may see as many as 5 new sarcoma patients in a week. Furthermore, it would be advantageous if patients were to receive all of their care (i.e. radiation and surgery) in one specialized sarcoma center, where the treating physicians are in close contact allowing for less disjointed care.

Recent advances in radiation treatment now allow for higher doses per treatment to be delivered safely. Reductions in the total duration of radiation treatment are now standard of care for breast and prostate cancer. Advances in the reduction of treatment duration for sarcomas are lacking.

We have now completed our initial analysis of the first 52 patients treated on this 5-day protocol, with encouraging results in terms of local control and toxicity(*6*). We chose a dose (30 Gy delivered over 5 fractions) that would be biologically equivalent to the conventional radiotherapy dose of 50 Gy delivered over 25 fractions. This was calculated based on the linear quadratic formula, where biological effect (E) is determined by the number of fractions (N) and size of the dose per fraction (d) using the formula $E = (d + \alpha\beta d2)Nd$. The α/β ratio for STS is presumed to be 4. The 5 fraction dose roughly equivalent to 50 Gy administered in 25 fractions is approximately 30 Gy (6 Gy x 5). The 5 fraction dose roughly equivalent to 28 Gy administered in 8 fractions (which is a regimen often used for patients receiving chemotherapy) is approximately 25 Gy (5 Gy x 5).

3.0 PATIENT SELECTION

3.1 Conditions for patient eligibility

3.1.1 Histologically confirmed soft tissue sarcoma of the extremity/trunk
3.1.2 Planning to undergo standard preoperative radiotherapy
3.1.3 Resectable primary lesion (patients with pre-existing metastasis will be included if their primary is still going to be resected)
3.1.4 Age ≥ 12
3.1.5 KPS ≥ 70 or ECOG of 0 - 2
3.1.6 If a woman is of childbearing potential, a negative serum or urine pregnancy test must be documented.

3.2 Conditions for patient ineligibility

3.2.1 Active treatment of a separate malignancy

3.2.2 History of prior irradiation to the area to be treated

4.0 REGISTRATION PROCEDURES

4.1 General guidelines

Patients seen at UCLA as new patients diagnosed with soft tissue sarcoma who are considering pre-operative radiation treatment will be informed of this clinical trial. The decision to participate will be voluntary. Eligibility will be determined at the time of consultation. The decision not to participate in the trial will not affect the delivery of standard treatment options.

4.2 Registration Process

An informed consent form will be given to the patient for review. Consent will be obtained after a clear and thorough discussion between the patient and the study investigator in the clinic. To register a patient, the research coordinator will obtain or complete:

- signed informed consent form;
- signed HIPAA authorization form;
- pathological confirmation of soft tissue sarcoma (UCLA pathology review not required);
- medical history;
- documentation of clinical examination;
- negative serum or urine pregnancy test for women of childbearing potential.

The patient will be enrolled on the study if all eligibility criteria are satisfied; otherwise, the patient will not be enrolled on the study.

5.0 TREATMENT PLAN

5.1 Radiation Simulation and Planning

Each patient will undergo radiation simulation and planning. A custom vacloc bag, alpha cradle, or equivalent immobilization device will be used. A CT and/or MRI simulation will be obtained with or without IV contrast, which is standard of care for any patient undergoing radiation therapy for soft tissue sarcomas.

The study investigator will be responsible for delineating the gross tumor volume (GTV) using the CT as well as MRI imaging (if available) performed as part of staging. Guidelines for contouring will be as per the currently open NRG trial for sarcomas. In general this suggests a margin of 3 cm in the superior and inferior directions on the gross tumor volume and a 1.5 cm radial expansion. This expansion should also include any suspicious edema as seen on T2 weighted MRI. This clinical target volume (CTV) will be cropped out of natural anatomic boundaries of spread, such as bone and uninvolved muscle compartments. The CTV will then be expanded to a planning treatment volume (PTV) using a 3-5 mm expansion.

A prescription dose of 6 Gy x 5 fractions (30 Gy) will be delivered to at least 95% of the PTV. No more than 10% of the PTV should receive greater than 120% of prescription dose, and areas receiving >110% of the prescription dose should be restricted to the GTV. Intensity modulated radiation therapy (IMRT) planning techniques may be used in order to minimize radiation dose to nearby organs at risk (OAR) but is not required.

Delineation of normal structures including the bone and skin will be performed and verified by the responsible study investigator. The radiation physicist or dosimetrist will optimize the treatment plan prior to approval for treatment. Dose volume histograms (DVH) and normal tissue constraint parameters specified below will be used to judge the plan quality and optimize PTV coverage with OAR sparing prior to approval.

5.2 Organs at Risk (OAR) Dose Constraints/Normal Tissue Constraints

Dose constraints will be used during radiation planning to minimize risk of toxicity. Dose constraint guidelines are shown below, and the treating physician will weigh meeting these constraints with any loss in coverage of the PTV. These constraints are designed as guidelines and failing to meet any of these constraints will not be considered a study deviation. Priority of constraints will depend on the precise anatomic location of the tumor.

Organs at risk:

- Total Skin within field (the superficial 5mm of tissue starting at the air/skin interface and encompassing the PTV): V12 Gy < 50%, V10Gy ≤ 150cc, D5cc ≤ 28-30 Gy
- Longitudinal strip of skin/subcutaneous tissue, defined for extremity tumors only as a 2cm thickness strip on the contralateral aspect of the extremity: V12Gy < 10%, D50% <12 Gy.
- Long bones (femur, humerus): maximum dose 31.5 Gy, D50cc≤28Gy
- Femoral or humeral head: V30Gy < 5cc, maximum <31.5 Gy
- Joints (knee, ankle, hip, shoulder, elbow, wrist): V50% ≤ 32 Gy
- Genitalia (e.g. vulva, perineum): V25Gy ≤ 10%
- Reproductive organs (testes, ovaries): mean (combined right & left) ≤ 2 Gy
- Spinal cord: max 30 Gy
- Chest wall: V30Gy<70cc
- Bowel: max 32 Gy, V30 Gy < 5cc
- Liver: V15Gy < 700cc
- Kidney (bilateral): V10Gy < 10%
- Heart: mean dose <8Gy
- Brachial plexus: max dose <33 Gy, D5cc ≤31.5 Gy

5.3 Treatment Verification and Delivery

Prior to each treatment delivery, daily image guidance will be used to verify the patient's treatment position and appropriate patient alignment. This can include but is not limited to kV imaging, MV imaging, cone beam CT image guidance, or MRI guidance.

Radiation will be delivered daily for 5 consecutive days with the exception of weekends and holidays. In instances where the radiation treatment week contains a holiday or scheduling availability is limited, two fractions of radiation may be given on the same day providing that the fractions are administered ≥ 6 hours apart (this is considered standard of care treatment). Treatment will be completed within a maximum of 12 days.

5.4 Patient Follow-up after Treatment

Patients will be seen in clinic within 3 months after the completion of radiation by either the radiation oncologist or the surgeon. After this, follow-up will be performed every 6 months after the time of surgery for the first 5 years after treatment (+/- 2 months). The patients will be followed both clinically and radiographically after treatment. CT or MRI will be performed of the primary site and CT of the chest every 6 months (+/- 2 months) for the first 2 years and then once at the end of year 3, or at the investigators' discretion. Whenever feasible and in the best interest of subjects, follow-up visits may occur via telemedicine.

Correlative translational research:

(1) Germline Biomarkers: the discovery of microRNAs (miRNAs) has led to an enormous advance in our understanding of human genetics. As their function and biology has been uncovered, it has become clear that miRNAs and their binding sites in the 3' untranslated region (3'UTR) of genes explains much of the mystery about the importance of inherited variability of non-coding regions of the genome. Through significant understanding of miRNAs, new germ-line genetic markers of cancer risk as well as predictive biomarkers of response to cancer therapy have rapidly emerged(*10, 11*). To collect germline information, whole blood or buccal swabs will be collected at baseline, during or after treatment.

(2) Immune profiling: Soft tissue sarcomas are known in some cases to respond to immune-based therapies (*12, 13*). These tumors are known to have a dense infiltrate of macrophages, which can have tumor-promoting characteristics. The presence of macrophages within the tumor microenvironment is known to be associated with worse prognosis across several malignancies(*14*). Likewise, radiotherapy (in particular hypofractionated radiotherapy), can result in the recruitment of macrophages to the tumor microenvironment, which can promote resistance to radiotherapy(*15*). Approaches that modulate macrophage infiltration and function are gaining clinical interest. To understand the impact of macrophages on prognosis and treatment response, and define potential therapeutic of targeting macrophages in soft tissue sarcomas, especially in

combination with hypofractionated radiotherapy, we plan to evaluate formalin-fixed paraffin-embedded tissue from pre-treatment biopsy for patients where this tissue is available. We will collect post-radiotherapy surgical specimens at the time of surgery, which will be used fresh, frozen, or formalin-fixed and paraffin-embedded for subsequent use. We will also collect ~10-20 mL whole blood in tubes containing anticoagulant (e.g. EDTA) prior to radiotherapy, during radiotherapy, after radiotherapy and after surgery, to evaluate dynamic changes in peripheral immune system to evaluate the impact of radiotherapy and surgical removal of the disease. Whole blood will be analyzed immediately by flow cytometry, and plasma and PBMC will be frozen for future analysis.

T cell based immunotherapies are an emerging therapy in the treatment of many malignancies, and there is interest in soft tissue sarcoma. This therapy may be predicated on the presence of nonsynonymous mutations within the tumor that are recognized by the immune system (neoepitopes), and the presence of functional T cells within the tumor microenvironment. New computational methods may allow for prediction of neoepitopes using next-generation sequencing of the tumor(*16*). Next-generation sequencing (NGS) of sarcomas has led to deeper understanding of disease categorization and subtype and revealed potential therapeutic targets(*17*). Tumor-specific T cells can be evaluated using deep sequencing of T cells isolated from the tumor. However, despite these tools, implications of sarcoma genomics on the anti-tumor immune response are not understood. To this aim, we will collect tumor specimens at the time of surgery for the isolation of immune cells from the tumor and for next generation sequencing of the tumor.

(3) Distant metastasis after local therapy for soft tissue sarcoma continues to be a clinical obstacle. Comparing the genomic landscape of primary and metastatic lesions may provide insight into the timeline of evolution of metastasis in sarcoma, and inform better adjuvant therapies. Thus, for patients who undergo surgery for relapsed or metastatic sites, we will also collect tumor specimens at the time of surgery to isolation of immune cells from the tumor and for next generation sequencing.

6.0 PHARMACEUTICAL INFORMATION

6.1 Investigational Agent or Device

Not applicable.

6.2 Availability

Not applicable.

6.3 Agent Ordering
Not applicable.

6.4 Agent Accountability
Not applicable.

7.0 ADVERSE EVENTS: LIST AND REPORTING REQUIREMENTS
Toxicity assessment will be performed using the National Cancer Institute Common Terminology Criteria for Adverse Events (NCI-CTCAE) Version 4.0 for early (≤ 3 months), and late (> 3 months) radiation toxicity. Dose limiting toxicity will be defined as any treatment-related grade 3 or higher toxicity in the following categories: skin and extremity. In addition, any other grade 4 or 5 toxicity attributed to the therapy will be reported immediately.

7.1 Treatment Toxicities

7.1.1 Constitutional symptoms
Constitutional symptoms that may be attributed to radiation therapy include fatigue, loss of appetite, nausea and vomiting. Patients will be seen on a weekly basis while they are receiving radiation therapy, per standard of care. Counseling and medications may be prescribed to alleviate these symptoms while the patient is on treatment. It is expected that symptoms will improve and resolve 2-4 weeks after completion of therapy.

7.1.2 Radiation-induced skin toxicity
Patients may develop acute skin toxicities which will be monitored during and after their treatment. Wound complications that occur as a result of skin toxicities will be monitored within 3 months after surgery and scored as a major wound complication per the criteria set forth in Appendix.

8.0 STUDY CALENDAR

	Pre-Treatment		Treatment		Follow-up		Relapse/Metastatic Site (if applicable)
	**Pre-Study**	**Pre-RT**	**RT**	**Surgery within 12 wks post radiation**	**Within 3 months from time of surgery(+/- 1 month**	**Every 6 months, up to 5 years (+/- 2 months) from time of surgery**	
**History & Physical**	X				x	x	
**Consent**	X						
**CT Chest**	X					X (every 6 months x 3 years) (+/- 2 months)	
**CT or MRI of primary site**	X					X (every 6 months x 3 years) (+/- 2 months)	
**Pregnancy test**	X						
**Tissue collection**				x			X
**Blood Collection**		X	X[1]	X*	X*	X[2]	X
**MRI/CT Simulation and RT Planning**		X					
**Radiotherapy**			X				
**Surgery**				X			
**Clinical Follow-up[6]**					X	X	
**Wound Assessment/Complication**					X[3]		
**RTOG/EORTC**					X	X	
**TESS**	X[4]					X[4]	
**MSTS**	X[5]					X[5]	
**CTCAEv4**					X	X	

[1] Serum study draw will occur whenever feasible, approximately Midpoint of radiation treatment (+/- 2 weeks), or at the discretion of the Investigator.
[2] Serum samples will be drawn whenever feasible, approximately at 6 month follow-up visits, (+/- 2 months), or at the discretion of the Investigator.
[3]Wound complications will be assessed for the first 120 days after the completion of surgery
[4] TESS questionnaires will be completed by the patient at baseline, approximately 12 months, 18 months and 24 months from the start of treatment. These may be administered electronically or via telemedicine.
[5] MSTS will be completed by the PI at baseline, approximately 12 months, 18 months and 24 months from the start of treatment
[6] Whenever feasible and in the best interest of the subject, follow-up visit may occur via telemedicine.
*annotates optional

Early Stopping Rules for Toxicity

Stopping rules of toxicity will be related to dose limiting toxicity as described. The trial will be stopped early if the acute wound toxicity is greater than 15% higher than that seen with standard fractionation in the NCIC randomized trial of pre-operative versus post-operative radiation of approximately 35%. If a single patient has more than one dose limiting toxicity, they will only be counted as one acceptable toxic event for this analysis.

9.0 DATA REPORTING/REGULATORY CONSIDERATIONS

9.1 Monitoring Plan

The JCCC DSMB meets monthly to review all SAE reports for trials overseen by the JCCC DSMB. All SAE reports, which have been filed since the previous meeting, are presented to the committee for review.

For trials overseen by the JCCC DSMB, the DSMB reviews all dose-limiting toxicities (DLTs) for dose-escalation studies. Protocol suspensions and re-opening of accrual to the next cohort based on DLT evaluation fall under the purview of the DSMB.

For all JCCC oncology trials and TRIO-US studies where the JCCC DSMB has primary oversight, all SAEs shall be reported to the JCCC DSMB in a timely manner [ten days, two days for a death] regardless of relationship and expectedness. The JCCC ORC will review all submissions and the ORC staff will enter the information into the JCCC Clinical Trials database. Reports are generated for full JCCC DSMB review. For trials where the JCCC DSMB has primary DSMB review responsibility, the DSMB requires that the PI generate cumulative adverse event reports for quarterly, biannual or annual review.

The DSMB reviews each SAE report and determines whether or not protocol modifications are warranted to ensure subject safety. In this review, prior occurrences of similar toxicity with the therapy under study are taken into consideration, as well as the severity of the event and the likelihood that it was related to a study drug. The DSMB may recommend no changes to the study if the event is expected or related to other causes such as the subject’s underlying condition. The DSMB may request an expert’s advice of another non-Principal Investigator with national experience to support their deliberations and decisions.

The JCCC DSMB has the authority to recommend to the UCLA IRB the immediate halt to a study (i.e., discontinuation of any further treatment of enrolled subjects and discontinuation of enrollment of new subjects) should there be any serious unexpected toxicity that warrants further investigation.

Requests for single subject exceptions/waivers from the approved study protocol, including out of window procedures and eligibility deviations, must be reviewed and approved by a member of the DSMB. Each trial is assigned a primary and secondary reviewer who is responsible for reviewing each exception/waiver request for that trial. Approvals and disapprovals of the request are sent to the Principal Investigator via email and copied to the UCLA IRB. Requests for single subject exceptions/waivers are made

by the Principal Investigator via email utilizing the "Single Subject Exception Request Form."

JCCC DSMB correspondences are addressed to the Principal Investigator and copied to the UCLA IRB. Minutes of the DSMB meetings are maintained in a computer file.

Confidentiality: Each member of the JCCC DSMB is responsible for maintaining strict confidentiality of the study data. Members will not share any study data or information about the study with any individual external to the JCCC DSMB or the statistical working group for the study. The DSMB members may contact the statistical working group directly with questions regarding the operational details associated with the data analysis and summary presentations. Communication of deliberations or recommendations of the JCCC DSMB, either written or oral, should not be made outside of the Committee or the statistical working group. Outcome results are strictly confidential and must not be divulged to any non-member of the JCCC DSMB except in those cases where DSMB is required to inform the UCLA IRB of its determinations. Disclosure of outcome results to the IRB must only occur with written approval of the DSMB. A member who believes he or she may have a potential intellectual or financial conflict of interest during the course of review of the data must inform the chairperson of the DSMB. In such case, the meeting minutes will record the disclosure of the potential conflict of interest and that the individual recuse himself from the discussions and abstains from voting on the DSMB decision.

The development of complications will be monitored. If rates of development of acute wound complications exceeds 50% of patients, the study will be terminated because it will be assumed that the regimen is toxic compared with standard pre-operative radiation therapy where wound complication rates are reported to be 43% based on the NCIC study.

Level of Risk of a Study

All interventional clinical trials undergo scientific review by the Internal Scientific Peer Review Committee (ISPRC) which requires that a Data and Safety Monitoring Plan is in place before a trial can be approved to begin. For trials overseen by the JCCC DSMB, the JCCC DSMB will determine the degree of risk of the study and will ensure that there are procedures in place to ensure the safety of the subjects that are enrolled in the trial. The intensity level of study oversight is determined by the risk category. Some of the factors that are considered when assigning the Level of Risk category include:

- A biostatistical design and appropriate procedures for proper data management so that the information collected can be properly validated.
- Appropriate Serious Adverse Event reporting procedures must be in place.
- The study duration must be appropriate and must be based on a realistic rate of enrollment.

- Data collection and data management must be adequate to verify and ensure subject eligibility.

Assignment of risk

Assigning risk ensures that the data and safety monitoring is based on the level of risk (low, medium, or high) to ensure that the data and safety monitoring activities are appropriate. Below are some of the criteria used to make a decision regarding the assignment of risk:

- Expected duration of the study based upon the estimated rate of enrollment.
- Type of study population (e.g., children, geriatric)
- The procedures used in the trial are commensurate with the degree of risk.
- Adequate data management systems in place and appropriate case report forms
- Proper serious adverse event reporting procedures in place
- Proper biostatistical design and data analysis procedures in place.

Level 2

- Compliance Officer meets with PI/Staff prior to study initiation to review regulatory requirements and operating system. Compliance Officer provides real-time monitoring to determine eligibility prior to enrollment onto the protocol.
- Real-time QA monitoring of the subjects and data collection occurs for all subjects entered onto the trial.
- Comprehensive QA auditing within the first year or first 10 subjects enrolled, whichever comes first. Subsequent audit frequency will be annually.
- Frequency of DSMB Summary Report is typically on a biannual basis or approximately every six months.

Monitoring and Auditing Activities

The compliance officer of the JCCC Office of Regulatory Compliance [ORC] will monitor and audit the clinical records for all human subjects enrolled onto JCCC trials overseen by the JCCC DSMB. The JCCC compliance officer will perform real time review of informed consent processes and the meeting of all inclusion and exclusion criteria and screening results at study entry. Active monitoring will offer the JCCC study teams prospective information that can be used to enhance the quality of research being performed contemporaneously. Auditing is a review of historic performance of the research effort and is performed on case report forms, regulatory files and source documents to measure the quality of the research effort in a retrospective manner.

9.2 Data Management and Data Security

The Radiation Oncology research staff will be responsible for the database records of study patients. The data will be kept on the research coordinator's firewall-protected computer, under password protection. Only the research team (study nurse, investigators, and project supporting staff) will have access to any identifiable subject information or other PHI. Case report forms will be used to record trial data and each subject will be assigned a code which is entered into the CRF. These case report forms will also include a place for recording adverse events.

10.0 STATISTICAL CONSIDERATIONS

10.1 Statistical considerations

1. Interim Reports for the Secondary Endpoint Grade ≥ 2 radiation morbidity (subcutaneous tissue fibrosis, joint stiffness, or edema) at 2 years

Interim reports will be prepared yearly until the results of the study are published. In general, the interim reports will contain information about patient accrual rate with projected completion dates, status of QA review and compliance rate of treatment per protocol, and the frequencies and severity of toxicity.

2. Estimation of Secondary Endpoints Related to the safety and efficacy

Cumulative incidence approach (K-M plots and COX proportional hazard modeling) will be used to estimate the local failure, regional failure, distant metastasis, progression-free survival, and overall survival rates.

10.2 Patient Accrual and Study Duration

10.2.1

The accrual of the initial cohort of the study (N=52) was completed enrollment on 6/11/2018. It is expected that the expansion cohort of the study protocol will complete accrual within 7 years after the initiation of the expansion cohort. To account for potential screening failures or participant dropouts, the total enrollment goal for the expansion cohort is 205 patients.

The study expansion will then continue for an additional 1 year to obtain follow-up data for the primary endpoint for the expansion phase of the study.

Study-related data will be stored for 5 years after termination of the study accrual and when all patients have completed follow-up procedures.

10.3 Analysis Plan

10.3.1 <u>Interim Reports</u>

Interim reports will be prepared yearly until the results of the study are published. In general, the interim reports will contain information about patient accrual rate with projected completion dates, status of QA review and compliance rate of treatment per protocol, and the frequencies and severity of toxicity.

11. REFERENCES

1. J. C. Yang *et al.*, Randomized prospective study of the benefit of adjuvant radiation therapy in the treatment of soft tissue sarcomas of the extremity. *Journal of Clinical Oncology* **16**, 197-203 (1998).
2. B. O'Sullivan *et al.*, Preoperative versus postoperative radiotherapy in soft-tissue sarcoma of the limbs: a randomised trial. *Lancet (London, England)* **359**, 2235-2241 (2002).
3. D. Wang *et al.*, Significant Reduction of Late Toxicities in Patients With Extremity Sarcoma Treated With Image-Guided Radiation Therapy to a Reduced Target Volume: Results of Radiation Therapy Oncology Group RTOG-0630 Trial. *J Clin Oncol* **33**, 2231-2238 (2015).
4. M. R. Folkert *et al.*, Comparison of local recurrence with conventional and intensity-modulated radiation therapy for primary soft-tissue sarcomas of the extremity. *J Clin Oncol* **32**, 3236-3241 (2014).
5. H. D. Thames, H. D. Suit, Tumor radioresponsiveness versus fractionation sensitivity. *International journal of radiation oncology, biology, physics* **12**, 687-691 (1986).
6. A. Kalbasi *et al.*, A Phase 2 Trial of Five-Day Neoadjuvant Radiation Therapy for Patients with High-Risk Primary Soft Tissue Sarcoma. *Clin Cancer Res*, (2020).
7. J. D. Pennington *et al.*, Long-term Outcomes With Ifosfamide-based Hypofractionated Preoperative Chemoradiotherapy for Extremity Soft Tissue Sarcomas. *American journal of clinical oncology*, 1 (2018).
8. D. G. LeBrun *et al.*, Predictors of Wound Complications following Radiation and Surgical Resection of Soft Tissue Sarcomas. *Sarcoma* **2017**, 1-7 (2017).
9. R. c. team, *Stats package (power.prop.test() function) in R: A language and environment for statistical computing.*, (Vienna, Austria: R Foundation for Statistical Computing, 2014).
10. L. J. Chin *et al.*, A SNP in a let-7 microRNA complementary site in the KRAS 3' untranslated region increases non-small cell lung cancer risk. *Cancer research* **68**, 8535-8540 (2008).
11. J. Weidhaas *et al.*, The KRAS-variant and treatment response in BATTLE-1. *J Clin Oncol* **32**, suppl; abstr 8135 (2014).
12. S. P. D'Angelo *et al.*, Nivolumab with or without ipilimumab treatment for metastatic sarcoma (Alliance A091401): two open-label, non-comparative, randomised, phase 2 trials. *The Lancet Oncology* **19**, 416-426 (2018).
13. H. A. Tawbi *et al.*, Pembrolizumab in advanced soft-tissue sarcoma and bone sarcoma (SARC028): a multicentre, two-cohort, single-arm, open-label, phase 2 trial. *The Lancet Oncology* **18**, 1493-1501 (2017).

14. B.-Z. Qian, J. W. Pollard, Macrophage diversity enhances tumor progression and metastasis. *Cell* **141**, 39-51 (2010).
15. A. Kalbasi *et al.*, Tumor-derived CCL2 mediates resistance to radiotherapy in pancreatic ductal adenocarcinoma. *Clinical Cancer Research* **[Epub ahea**, (2016).
16. H. Hackl, P. Charoentong, F. Finotello, Z. Trajanoski, Computational genomics tools for dissecting tumour-immune cell interactions. *Nature reviews. Genetics* **17**, 441-458 (2016).
17. J. Barretina *et al.*, Subtype-specific genomic alterations define new targets for soft-tissue sarcoma therapy. *Nature Genetics* **42**, 715-721 (2010).

APPENDIX

1. Criteria for major wound complication
2. Late radiation toxicity grading
3. CTCAEv4 Criteria
4. Toronto Extremity Salvage Score (TESS) Questionnaire (patient-reported)
5. Musculoskeletal Tumor Society (MSTS) Score (physician-reported)

Criteria for a major wound complication

1) Secondary operations required for wound treatment (debridement, secondary closure procedures such as rotation flaps, free flaps, or skin grafts)

2) Readmission to hospital for wound care

3) Invasive procedures required for wound care (drainage of hematoma, seroma or infected wound collection, use of vacuum-assisted closure therapy) to an area of the wound measuring at least 2 cm in length

4) Prolonged dressing changes, including packing of the wound for greater than 6 weeks from wound breakdown

5) Repeat surgery for revision of split thickness skin graft or requirement for wet dressings for longer than 4 weeks. (it is permissible for a patient to protect a totally epithelialized skin graft with a dry dressing without declaring a major wound complication).

Criteria adapted from: O'Sullivan B, et al. Phase 2 study of preoperative image-guided intensity-modulated radiation therapy to reduce wound and combined modality morbidities in lower extremity soft tissue soft tissue sarcoma. Cancer. 2012.

Late radiation toxicity grading

	Grade				
Late Radiation Toxicity	0	1	2	3	4
RTOG/EORTC criteria<br><br>Subcutaneous fibrosis<br><br>Joint stiffness	 <br>None<br><br>None	 <br>Slight fibrosis; subcutaneous fat loss<br><br>Mild stiffness, slight range of motion loss	 <br>Moderate fibrosis; slight field contracture<br><br>Moderate stiffness, pain, range of motion loss	 <br>Severe fibrosis; field contracture > 10%<br><br>Severe stiffness; pain, range of motion loss	 <br>Necrosis<br><br>Necrosis; complete fixation
Stern's scale Edema	None	Mild, but definite swelling	Moderate	Severe (considerable swelling)	Very severe (skin shiny and tight with or without skin cracking)

CTCAEv4 Criteria that will be assessed

Constitutional
Fatigue
Weight loss

Skin
Skin hyperpigmentation
Skin induration
Radiation dermatitis
Desquamating rash
Dermatologic radiation recall reaction

Lymphatics
Limb edema

Musculoskeletal/soft tissue
Joint disorder
Seroma
Abnormal gait

Neurology
Peripheral sensory neuropathy
Peripheral motor neuropathy

Pain
In extremity
Skin
