# Supplementary material for: Five-Day Preoperative Radiation Therapy for Patients With High-Risk Soft Tissue Sarcoma: A Nonrandomized Clinical Trial
Source: JAMA Netw Open. 2025 Dec 17;8(12):e2550195. doi: 10.1001/jamanetworkopen.2025.50195 (PMC12712729; doi:10.1001/jamanetworkopen.2025.50195)
Supplement: Supplement 2. — eTable 1. Dosimetric Parameters for Radiation Planning for the Original Cohort eTable 2. Dosimetric Parameters for Radiation Planning for the Expansion Cohort eTable 3. Multiple Logistic Regression Analysis for Associations With Experiencing Grade 2 or Higher Radiation-Associated Toxicities eTable 4. Multiple Logistic Regression Analysis for Associations With Experiencing a Major Wound Complication eTable 5. Cox Regression Analysis for Associations With Experiencing Local Failure [file jamanetwopen-e2550195-s002.pdf]

Supplemental Online Content

Nikitas J, Kendal JK, Savjani RR, et al. Five-day preoperative radiation therapy for patients with high-risk soft tissue sarcoma. *JAMA Netw Open*. 2025;8(12):e2550195. doi:10.1001/jamanetworkopen.2025.50195

eTable 1. Dosimetric Parameters for Radiation Planning for the Original Cohort
eTable 2. Dosimetric Parameters for Radiation Planning for the Expansion Cohort
eTable 3. Multiple Logistic Regression Analysis for Associations With Experiencing Grade 2 or Higher Radiation-Associated Toxicities
eTable 4. Multiple Logistic Regression Analysis for Associations With Experiencing a Major Wound Complication
eTable 5. Cox Regression Analysis for Associations With Experiencing Local Failure

This supplemental material has been provided by the authors to give readers additional information about their work.

© 2025 Nikitas J et al. *JAMA Network Open*.

eTable 1. Dosimetric Parameters for Radiation Planning for the Original Cohort

**Organ-at-Risk**	**Dose Constraint**
Planning target volume	V110% ≤ 10%
Total Skin within field (the superficial 5 mm of tissue starting at the air/skin interface and encompassing the planning target volume)	V12 Gy ≤ 50%
Longitudinal strip of skin/subcutaneous tissue (defined for extremity tumors only as a 2 cm thickness strip on the contralateral aspect of the extremity)	V12Gy < 10%
Long bones (i.e., femur or humerus)	V30Gy ≤ 50%
Femoral or humeral head	V30Gy < 5cc<br>Dmax ≤ 33 Gy
Spinal cord	Dmax < 30 Gy
Chest wall	V30Gy ≤ 70cc
Bowel	V32 Gy ≤ 5cc
Liver	V15Gy ≤ 700cc
Kidney (bilateral)	V10Gy ≤ 10%

© 2025 Nikitas J et al. *JAMA Network Open*.

eTable 2. Dosimetric Parameters for Radiation Planning for the Expansion Cohort

**Organ-at-Risk**	**Dose Constraint**
Total Skin within field (the superficial 5 mm of tissue starting at the air/skin interface and encompassing the planning target volume)	V12 Gy < 50%<br>V10Gy ≤ 150cc<br>D5cc ≤ 28-30 Gy
Longitudinal strip of skin/subcutaneous tissue (defined for extremity tumors only as a 2 cm thickness strip on the contralateral aspect of the extremity)	V12Gy < 10%<br>D50% <12 Gy
Long bones (i.e., femur or humerus)	Dmax < 31.5 Gy<br>D50cc ≤ 28Gy
Femoral or humeral head	V30Gy < 5cc<br>Dmax < 31.5 Gy
Joints (i.e., knee, ankle, hip, shoulder, elbow, or wrist)	V50% ≤ 32 Gy
Genitalia (e.g., vulva or perineum)	V25Gy ≤ 10%
Reproductive organs (i.e., testes or ovaries)	Dmean (combined right & left) ≤ 2 Gy
Spinal cord	Dmax < 30 Gy
Chest wall	V30Gy < 70cc
Bowel	Dmax < 32 Gy<br>V30 Gy < 5cc
Liver	V15Gy < 700cc
Kidney (bilateral)	V10Gy < 10%
Heart	Dmean < 8Gy
Brachial plexus	Dmax < 33 Gy<br>D5cc ≤ 31.5 Gy

© 2025 Nikitas J et al. *JAMA Network Open*.

eTable 3. Multiple Logistic Regression Analysis for Associations With Experiencing Grade 2 or Higher Radiation-Associated Toxicities

**Patient Characteristics**	**Odds Ratio for Grade ≥2 Radiation-Associated Toxicities (95% confidence interval)**	***p*-value**
Involvement of the adductor compartment (vs no involvement)	0.71 (0.11-3.33)	0.68
Involvement of the elbows or knees (vs no involvement)	1.20 (0.056-9.86)	0.88
Tumor size	1.17 (1.06-1.32)	**0.0037**
Use of 3D-CRT/electron radiotherapy (vs IMRT)	2.51 (0.32-14.58)	0.32

Abbreviations: IMRT = intensity-modulated radiotherapy; 3D-CRT = 3-dimensional conformal radiotherapy.

© 2025 Nikitas J et al. *JAMA Network Open*.

eTable 4. Multiple Logistic Regression Analysis for Associations With Experiencing a Major Wound Complication

**Patient Characteristics**	**Odds Ratio for Major Wound Complication (95% confidence interval)**	***p*-value**
Involvement of the adductor compartment (vs no involvement)	2.28 (0.65-8.17)	0.19
Involvement of the elbows or knees (vs no involvement)	0.25 (0.011-2.30)	0.27
Diabetes history (vs no history)	6.09 (1.39-27.11)	**0.015**
Current smoking history (vs no history)	3.95 (0.81-19.63)	0.086
Local tissue advancement flap with initial surgery (vs primary closure)	14.33 (4.81-48.88)	**<0.0001**
Vascularized flap with initial surgery (vs primary closure)	2.09 (0.087-21.52)	0.57

© 2025 Nikitas J et al. *JAMA Network Open*.

eTable 5. Cox Regression Analysis for Associations With Experiencing Local Failure

**Clinical Characteristics**	**Hazard Ratio for Local Failure (95% confidence interval)**	***p*-value**
Positive margin	4.81 (1.07-21.25)	**0.033**
Tumor size	1.04 (0.93-1.15)	0.40
Tumor grade	1.13 (0.38-3.98)	0.84
History of prior resection	1.82 (0.24-9.98)	0.51

© 2025 Nikitas J et al. *JAMA Network Open*.
