# Supplementary material for: Five-Day Preoperative Radiation Therapy for Patients With High-Risk Soft Tissue Sarcoma: A Nonrandomized Clinical Trial
Source: JAMA Netw Open. 2025 Dec 17;8(12):e2550195. doi: 10.1001/jamanetworkopen.2025.50195 (PMC12712729; doi:10.1001/jamanetworkopen.2025.50195)
Supplement: Supplement 3. — Data Sharing Statement [file jamanetwopen-e2550195-s003.pdf]

Data Sharing Statement

Nikitas. Five-Day Preoperative Radiation Therapy for Patients With High-Risk Soft Tissue Sarcoma. *JAMA Netw Open*. Published December 17, 2025.
doi:10.1001/jamanetworkopen.2025.50195

Data
Additional Information: ClinicalTrials.gov Identifier: NCT02701153
Data available: Yes
Data types: Other (please specify)
Additional Information: per study guidelines
How to access data: per study guidelines
When available: With publication

Supporting Documents
Document types: Other (please specify)
Additional Information: per study guidelines
How to access documents: per study guidelines
When available: With publication

Additional Information
Who can access the data: researchers whose proposed use of the data has been approved
Types of analyses: per study guidelines
Mechanisms of data availability: with investigator support
